# Prognostic Significance of the Modified H2FPEF Score in Patients with Severe Primary Mitral Regurgitation

**DOI:** 10.3390/jcdd13090439

**Published:** 2026-09-07

**Authors:** Hon Jen Wong, Tze Siang Koh, Zong Rui Bai, Jessele Lai, Nicholas L. X. Gao, Beth S. Y. Lim, Norman H. Y. Lin, Yao Hao Teo, Tony Y. W. Li, Vinay B. Panday, Raymond C. C. Wong, Tiong Cheng Yeo, Kian Keong Poh, William K. F. Kong, Ching-Hui Sia

**Affiliations:** 1 Department of Medicine, Yong Loo Lin School of Medicine, National University of Singapore, Singapore 117599, Singapore; wong_hon_jen@u.nus.edu (H.J.W.); e1123328@u.nus.edu (T.S.K.);; 2 Department of Cardiology, National University Heart Centre Singapore, Singapore 119228, Singapore

**Keywords:** H2FPEF score, mitral regurgitation, risk stratification, prognosis

## Abstract

Objectives: Severe mitral regurgitation (MR) has been associated with significant morbidity and mortality, necessitating effective risk stratification. The H2FPEF score has demonstrated prognostic value in other cardiac disorders. This study evaluates the prognostic utility of a modified version of the H2FPEF score for severe MR with preserved ejection fraction. Methods and Results: Patients from a tertiary cardiovascular referral center with consecutive index echocardiographic diagnoses of severe MR were included. We excluded patients with secondary MR, reduced LVEF (<50%) and/or missing information for modified H2FPEF score calculation. A modified H2FPEF score was utilized in view of differing obesity cutoffs in our Asian population (BMI > 25 kg/m^2^) as opposed to the original cutoff (BMI > 30 kg/m^2^). Patients were stratified based on the modified score into low (0–1), intermediate (2–5), and high (6–9) groups. The primary endpoint, a composite of all-cause mortality and first heart failure hospitalization (HFH), was analyzed using multivariate Cox regression. A total of 388 patients (low 57 [14.7%]; intermediate 218 [56.2%]; high 113 [29.1%] modified H2FPEF score) were included with a median follow-up duration of 4.53 years (interquartile range 2.61 to 6.60). Patients with a high modified H2FPEF score were older, more likely to have hyperlipidemia, diabetes, end-stage renal disease, higher NYHA class, and lower LVEF. Malay ethnicity, hyperlipidemia, diabetes, end-stage renal disease and a higher indexed left atrial diameter were independently associated with greater modified H2FPEF score. High and intermediate modified H2FPEF scores were associated with the primary endpoint (HR 9.17, 95% CI 2.15–39.1 and 6.71, 95% CI 1.62–27.9 respectively) versus low scores. However, a Fine–Gray regression showed no difference in subdistribution hazards of first HFH between high and intermediate scores. Conclusions: The modified H2FPEF score may facilitate risk stratification for all-cause mortality and first HFH in patients with severe primary MR and preserved ejection fraction.

## 1. Introduction

Mitral regurgitation (MR) is the most prevalent cause of valvular heart disease globally [1]. The prognosis of severe MR is poor, with long-term mortality estimated to be about 2.36-fold greater than those without MR [2]. Furthermore, severe MR appears to portend poorer survival despite optimal medical therapy [3]. A key challenge lies in frequent preservation of left ventricular ejection fraction (EF) in MR [4], which often results in an asymptomatic presentation [5]. This lack of symptoms can delay clinical diagnosis and treatment and obscure the true severity of disease [6]. This underscores the need for an objective risk stratification tool to help prognosticate and guide clinical decision making. Although individual clinical, echocardiographic, and biomarker parameters such as natriuretic peptides have established prognostic value in severe MR, there remains a lack of simple, readily applicable composite risk scores for routine prognostication.

The H2FPEF score is derived from readily available clinical and echocardiographic data. It was originally designed to assist in the diagnosis of heart failure with preserved ejection fraction (HFpEF) [7]. It has also demonstrated prognostic value in patients with hypertrophic cardiomyopathy [8] and in those undergoing transcatheter aortic valve implantation for severe aortic stenosis [9]. Both conditions can lead to progressive diastolic dysfunction, in part mediated by reduced left ventricular compliance.

Given that severe MR similarly predisposes to diastolic impairment, the H2FPEF score may have relevance in this population. Its individual components such as elderly age, elevated pulmonary artery systolic pressure (PASP), atrial fibrillation and elevated E/e’ ratio could be relevant to MR. However, to date, its application in patients with severe MR remains unexamined. Hence, this study seeks to evaluate the utility of the H2FPEF score for prognostication and risk stratification in patients with severe MR, while modifying its obesity component to fit Asian criteria. Such an approach may facilitate early identification of high-risk patients, guide interventional decision-making processes, and improve patient outcomes.

## 2. Materials and Methods

We performed a retrospective study of patients with consecutive index echocardiographic diagnoses of severe MR from 2010 to 2020 at a tertiary cardiovascular referral center in Singapore. A cohort of 721 patients was initially identified, with baseline, clinical and echocardiographic data extracted from their electronic medical records. Exclusion criteria included patients with secondary MR (Supplementary Figure S1), missing data for H2FPEF score calculation, and LVEF < 50% as the cutoff for HFpEF as per 2022 AHA/ACC/HFSA Guideline for the Management of Heart Failure [10]. The local institutional review board granted an exemption for written consent from participants for this study, as it utilized de-identified data (National Healthcare Group Domain Specific Review Board: 2021/00603). The study was conducted in accordance with the Declaration of Helsinki [11].

Transthoracic echocardiography was performed using commercially available ultrasound machines from Philips Medical Systems (Andover, MA, USA) and GE Healthcare (Chicago, IL, USA). All images were stored digitally and subsequently analyzed using proprietary software (EchoPAC 202; GE Vingmed Ultrasound, Horten, Norway). Two-dimensional, color, spectral continuous and pulse-wave Doppler images were acquired via the parasternal, apical and subcostal views. From the parasternal long-axis view, left ventricular (LV) end-diastolic diameter and left atrial diameter were measured [12]. The maximal LV end-diastolic wall thickness was assessed in the parasternal short-axis view at the basal, mid, and apical levels. LV ejection fraction (LVEF) was calculated using Simpson’s biplane method from the apical four-chamber and two-chamber views [12]. Continuous-wave Doppler was used to determine the LV outflow tract peak gradient at rest or during Valsalva maneuver. LV diastolic function was assessed by pulsed-wave Doppler recordings of transmitral flow at the mitral leaflet tips to measure peak early (E) and late (A) diastolic velocities [13]. Tissue Doppler imaging of the medial and lateral mitral annulus was used to measure e’, and averaged to calculate the E/e’ [13]. Systolic pulmonary artery pressure was estimated from the peak velocity of the tricuspid regurgitant jet using the Bernoulli equation, incorporating right atrial pressure derived from the inspiratory collapse and the diameter of the inferior vena cava [12]. Mitral regurgitation and severity were evaluated with a multiparametric approach [14].

The modified H2FPEF score was calculated using six variables [7]: obesity as defined by Asian criteria [15] (BMI > 25 kg/m^2^) as opposed to the WHO criteria (BMI > 30 kg/m^2^) in view of the Asian population included in this study, hypertension, atrial fibrillation, pulmonary hypertension as defined by PASP > 35 mmHg, age > 60 years, and elevated filling pressures indicated by E/e’ > 9. They were assigned equal weighting (1 point), except obesity (2 points) and atrial fibrillation (3 points). Patients were stratified into three groups based on their modified H2FPEF score: low (0–1 points), intermediate (2–5 points), and high (6–9 points) [8].

The primary endpoint was a composite of all-cause mortality and first heart failure hospitalization (HFH). Secondary endpoints included all-cause mortality and first HFH separately. The occurrence and date of death was obtained during review of the electronic medical records that are linked to the National Death Registry.

Baseline, clinical and echocardiographic characteristics of patients were presented as means with standard deviations (SD) for continuous variables and as frequencies and percentages for categorical variables. Between-group comparisons were conducted using Fisher’s exact test for categorical variables. The Kruskal–Wallis test was used for continuous variables. The median follow-up duration was derived via the reverse Kaplan–Meier estimator. Univariate ordinal logistic regression was used to evaluate the variables associated with the modified H2FPEF score. Significant variables were subsequently included in the multivariate model. The Brant test was used to assess the validity of the proportional odds assumption for the ordinal logistic regression models utilized. Time-to-mortality data were graphically represented using the Kaplan–Meier method. We assessed the association of low, intermediate and high modified H2FPEF scores with the primary endpoint via Cox proportional hazards regression. Variables deemed clinically significant based on the opinion of institutional experts were included in the multivariable Cox model presented [7,8,16]. We adjusted for sex, diabetes mellitus, hyperlipidemia, NYHA functional classification, end-stage renal disease, LVEF and indexed left ventricular end-systolic diameter. The proportional hazards assumption was evaluated through the Grambsch–Therneau test for a non-zero slope with linear regression of scaled Schoenfeld residuals [17]. Cumulative incidence curves for HFH and all-cause mortality were generated. The comparative hazards of HFH between high and intermediate modified H2FPEF scores were analyzed with the Fine–Gray subdistribution hazards model to account for all-cause mortality as a competing risk [18]. A similar multivariable model was utilized. Sensitivity analysis was performed by excluding patients who were recorded to undergo mitral valve intervention. Missing data were addressed via complete case analysis, incorporating only patients without missing relevant values. We excluded variables with >10% missing data [19]. Statistical analysis was performed using R statistical software Version 4.3.1, with statistical significance set at *p* < 0.05.

## 3. Results

**Figure 1:**
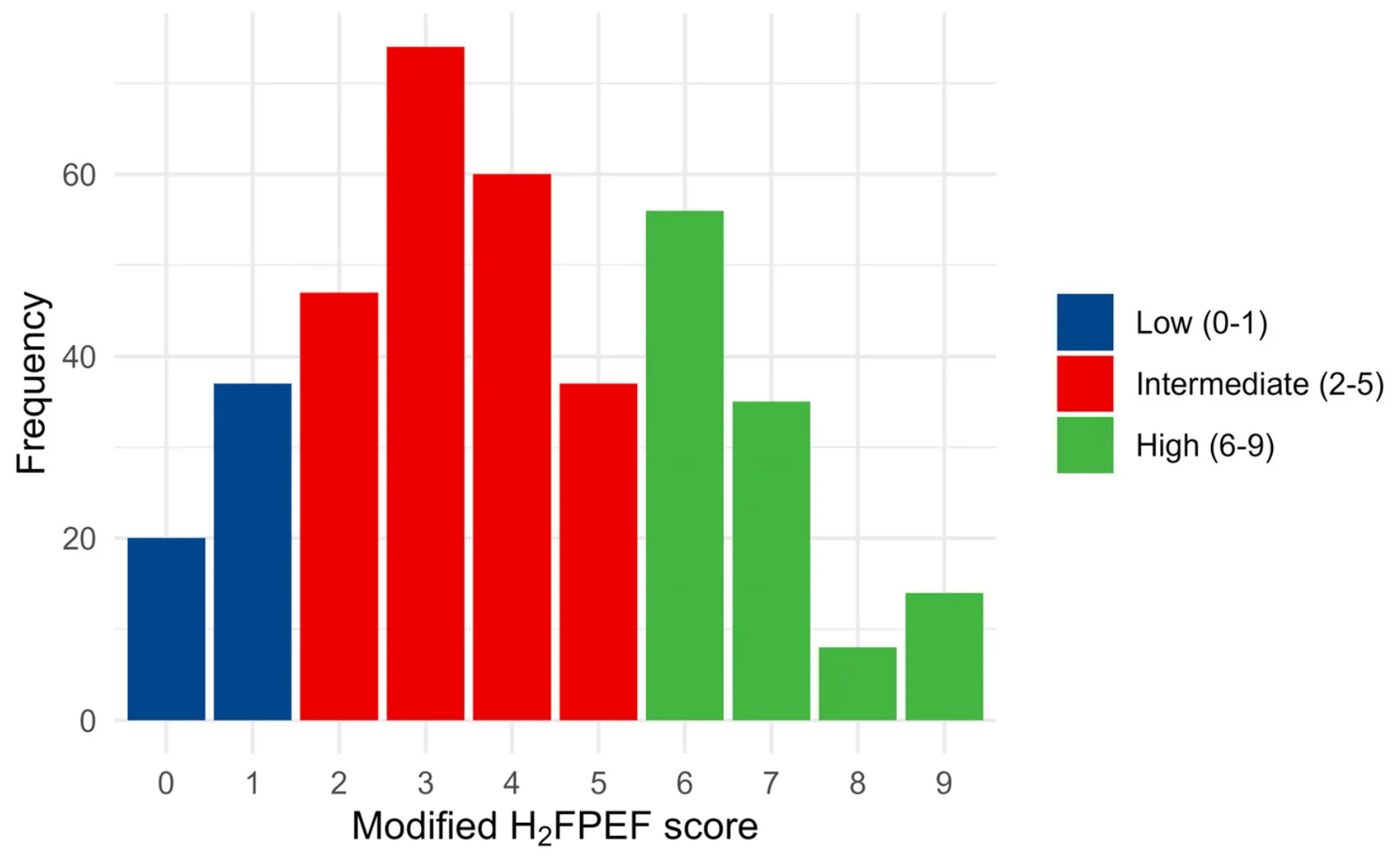
Distribution of modified H2FPEF scores.

We included 388 patients with severe MR and preserved ejection fraction (pEF) in the analysis. The mean modified H2FPEF score was 3.99 (SD 2.26) [Figure 1]. Baseline and clinical characteristics are summarized in Table 1. The mean age was 64 years (SD 15) and 247 (64%) patients were male. A total of 57 (14.7%) patients had a low modified H2FPEF score (0–1), 218 (56.2%) patients had an intermediate modified H2FPEF score (2–5) and 113 (29.1%) patients had a high modified H2FPEF score (6–9) [Table 1]. Patients with a high modified H2FPEF score were more likely to have DM, hypertension, hyperlipidemia, heart failure and ESRD versus those with an intermediate or low modified H2FPEF score. They also exhibited a greater symptom burden, as reflected by their higher NYHA classification. Patients with a high modified H2FPEF score tended to be prescribed more drugs, including beta-blockers, diuretics, statins and anticoagulants as compared to those with lower scores.

### 3.1. Echocardiographic Characteristics

Patients with high modified H2FPEF scores had lower LVEF compared to those with intermediate and low modified H2FPEF scores (Table 2). These patients were more likely to have elevated right ventricular peak pressure gradients, greater indexed left ventricular masses, and larger indexed left atrial diameters (LADi). They also demonstrated greater interventricular septal thickness in diastole, higher left ventricular posterior wall diameter in diastole, and greater relative wall thickness. However, they had comparable absolute and indexed left ventricular diameters and volumes, absolute left ventricular masses, MR effective regurgitation orifice areas, regurgitant volumes and regurgitant fractions versus those with intermediate and low modified H2FPEF scores.

### 3.2. Variables Associated with an Increasing Modified H2FPEF Score

We performed ordinal logistic regression (Table 3) to evaluate potential associations with a higher modified H2FPEF score. In the univariate analysis, male sex, Malay ethnicity, DM, HLD, ESRD, NYHA class III/IV, LVEF, LADi and LV mass index were associated with a higher modified H2FPEF score. These variables were included in the multivariate model, which showed a significant association of Malay ethnicity, DM, HLD, ESRD and LADi with an increasing modified H2FPEF score. The proportional odds assumption was not violated (Brant test, omnibus test statistic *p* = 1.00).

### 3.3. Association of the Modified H2FPEF Score with Study Endpoints

**Figure 2:**
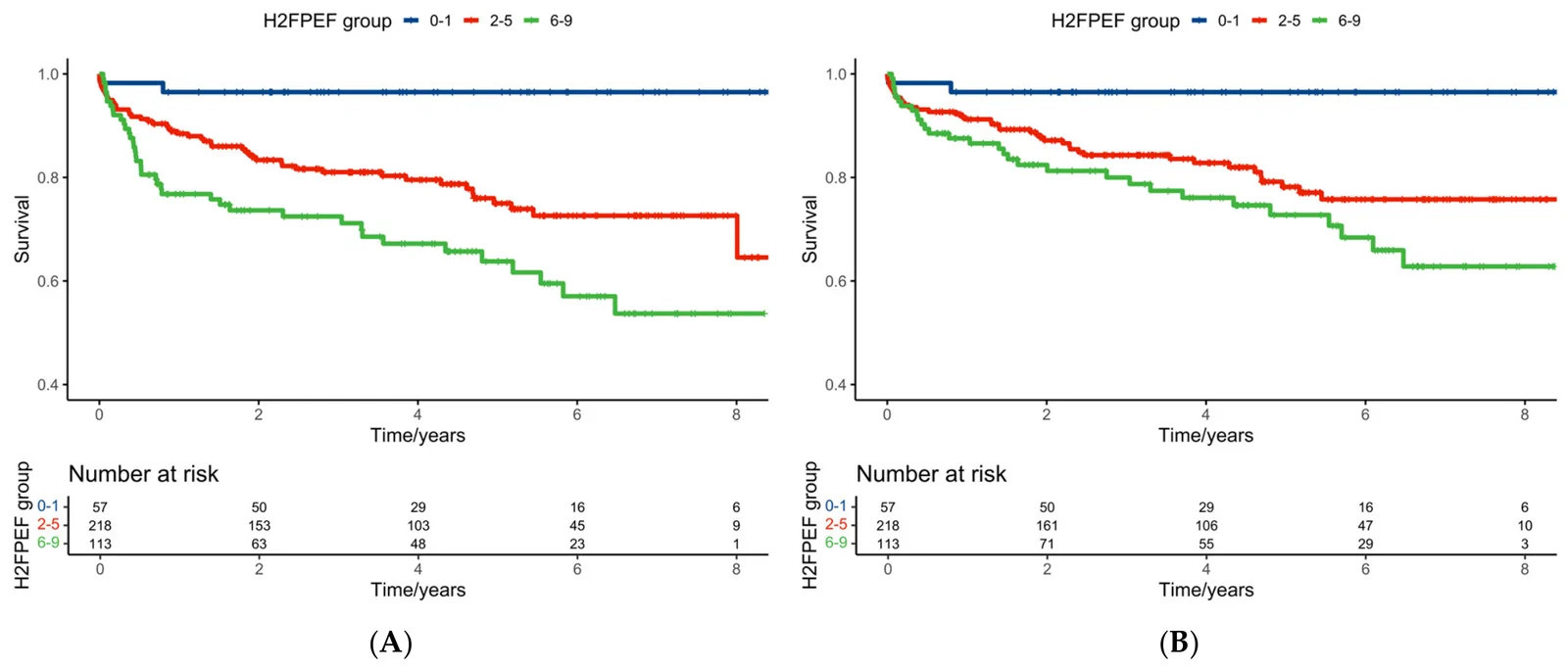
Kaplan–Meier curves for (**A**) the primary composite outcome and (**B**) all-cause mortality.

Over a median follow-up duration of 4.53 years (interquartile range 2.61 to 6.60), the primary composite endpoint occurred in 91 patients (23.5%). A total of 73 patients (18.8%) experienced all-cause mortality, and 33 (8.5%) had a first HFH. The Kaplan–Meier curve (Figure 2) demonstrated the poorest survival free from the primary composite endpoint for those with high modified H2FPEF scores followed by intermediate modified H2FPEF scores. After adjustment, an intermediate and high modified H2FPEF score were associated with an increased risk for the primary composite endpoint when compared to a low modified H2FPEF score (Supplemental Table S1). These associations persisted even after adjustment for differences in baseline, clinical and echocardiographic characteristics (intermediate score: HR 6.71, 95% CI 1.62–27.9; and high score: HR 9.17, 95% CI 2.15–39.1). Of note, the wide confidence intervals could reflect the small size of the low score group and limited number of HFH events. Sensitivity analysis excluding patients who underwent mitral valve intervention (Supplementary Table S2) affirmed the independent association of an intermediate or high modified H2FPEF score with the primary composite endpoint.

As for the secondary endpoint of all-cause mortality, patients with intermediate (HR 5.81, 95% CI 1.39–24.3) and high (HR 6.70, 95% CI 1.55–29.0) modified H2FPEF scores demonstrated a greater risk versus patients with low modified H2FPEF scores after adjustment (Supplemental Table S3). The Kaplan–Meier curve similarly demonstrated poorest survival for high modified H2FPEF scores (Figure 2). There was no violation of the proportional hazards assumption across all Cox regression models (all *p* > 0.05) (Supplementary Table S1–S3).

**Figure 3:**
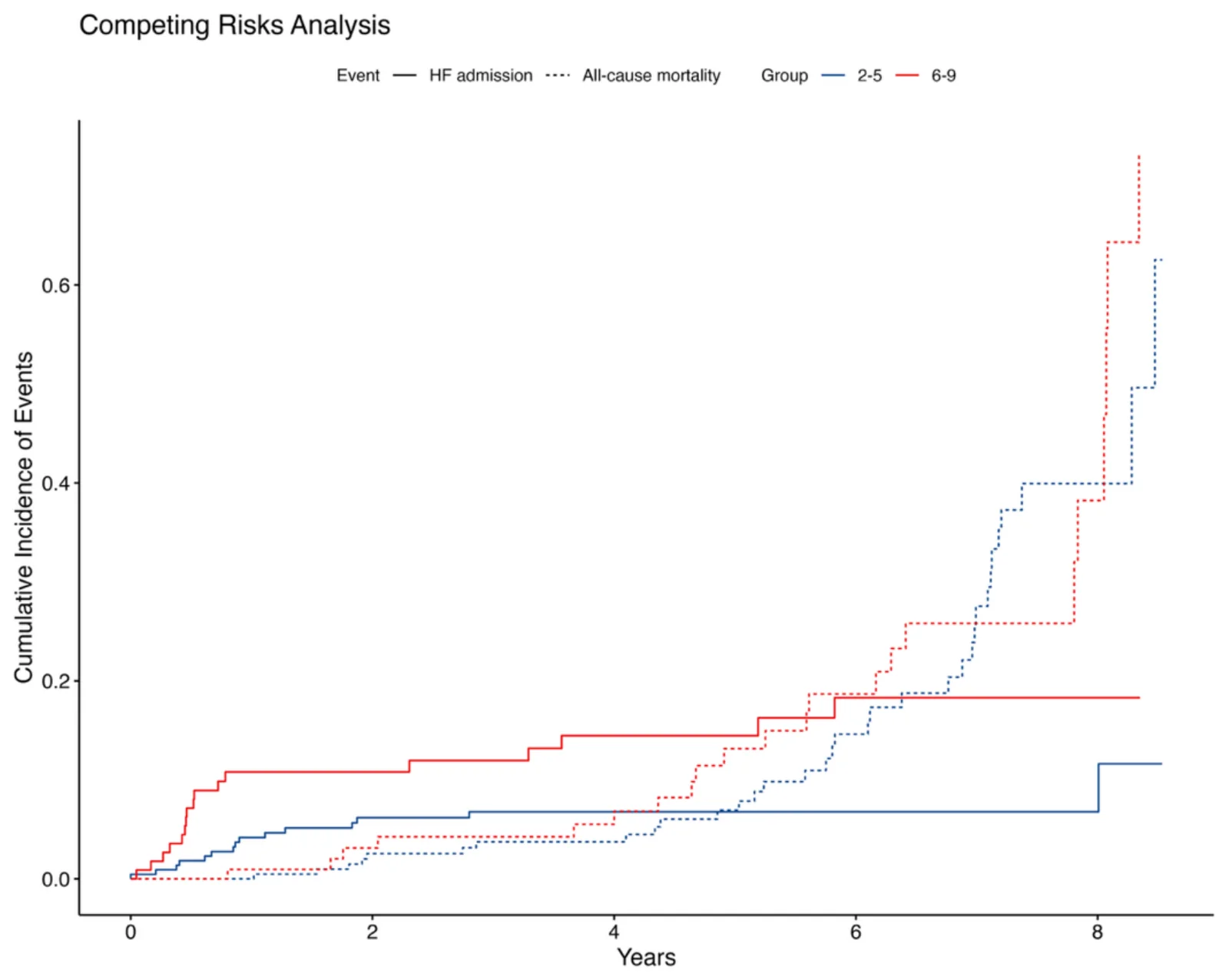
Cumulative Incidence Curve for First Heart Failure Admission with Death as Competing Risk in Patients with High and Intermediate H2FPEF scores (low H2FPEF scores omitted in view of zero first heart failure hospitalization events).

There were zero first HFH events across the entire duration of follow-up in patients with low modified H2FPEF scores. Hence, in the cumulative incidence curve and Fine–Gray model evaluating the subdistribution hazard of first HFH, we compared patients with high versus intermediate modified H2FPEF scores. The incidence of first HFH throughout the follow-up duration in patients with high scores was consistently greater than those with intermediate scores. The cumulative incidence of death appeared similar until the fourth year from index diagnosis (Figure 3). However, there was no significant difference in the risk of first HFH between high and intermediate scores (subdistribution HR 1.79, 95% CI 0.89–3.58, Supplementary Table S4).

## 4. Discussion

In this retrospective cohort study of patients with severe MR, we found that patients with a high modified H2FPEF score had a higher burden of comorbidities and were more likely to be symptomatic at presentation. They also demonstrated higher indexed left ventricular mass and larger indexed left atrial diameters, likely reflecting more advanced structural remodeling processes. The presence of diabetes, hyperlipidemia, end-stage renal disease and an increased indexed left atrial diameter were independently associated with a greater modified H2FPEF score. A high or intermediate modified H2FPEF score were significantly associated with a greater risk a composite of all-cause mortality and first HFH when compared to a low modified H2FPEF score, even after adjustment. However, there was no statistically significant difference in the risk of first HFH between high and intermediate scores, possibly reflecting limited power, after accounting for all-cause mortality as competing risk.

To the authors’ knowledge, this is the first study evaluating the modified H2FPEF score in patients with severe MR and pEF. Notably, our findings relating to its prognostic utility are largely congruent to that reported in other patient populations. A study evaluating the use of the modified H2FPEF score for risk stratification in patients with hypertrophic cardiomyopathy found that patients with a higher score presented with more symptoms and comorbidities, and lower LVEF [8]. A high modified H2FPEF score was likewise associated with a composite of all-cause mortality and HFH [8]. Akao et al. [9] demonstrated that a high modified H2FPEF score was independently associated with a similar composite outcome in patients receiving transcatheter aortic valve replacement.

The H2FPEF score was originally developed to assist in the identification of heart failure with preserved ejection fraction in patients with unexplained exertional dyspnea [7]. While it is now commonly used in clinical practice, it has not been validated for use in patients with severe MR. Nonetheless, the individual components of the H2FPEF score could be relevant in severe MR. For example, regurgitant flow into the left atrium results in volume overload and elevated left atrial pressure. This can lead to an increase in early diastolic peak mitral inflow velocity (E). The left atrial volume overload translates into left ventricular volume overload. Over time, this may contribute to left ventricular remodeling and impaired diastolic relaxation, reflected by a reduction in early diastolic mitral annular velocity (e’). An elevated E/e’ ratio, a component of the H2FPEF score, thus serves as an effective non-invasive surrogate marker of elevated left ventricular pressures often seen in severe MR, and may indicate a heightened risk for heart failure hospitalizations [20]. Of note, a higher indexed left ventricular mass, likely a consequence of left ventricular remodeling, was associated with a higher modified H2FPEF score [21].

In addition, the elevated left atrial pressures observed in severe MR are transmitted retrogradely into the pulmonary circulation, resulting in pulmonary hypertension [22]. A non-invasive means of assessing this is through the measurement of pulmonary artery systolic pressures via echocardiography [23]. Choudhary et al. [23] demonstrated that an elevated pulmonary artery systolic pressure is a predictor of heart failure and is associated with an increased risk of HFHs, particularly in patients with pEF [23]. The study also highlighted the clinical utility of PASP in screening of patients with cardiovascular comorbidities [23]. Hence, an elevated pulmonary artery systolic pressure, a component of the H2FPEF score, is of prognostic relevance in patients with severe MR and pEF.

Atrial fibrillation is strongly associated with left atrial dilation, which frequently develops as a consequence of MR [21]. In our study, we observed that an increased indexed left atrial diameter was significantly associated with a higher modified H2FPEF score, which includes the presence of atrial fibrillation as a component. Therefore, the presence of atrial fibrillation may reflect more advanced structural changes in severe MR, and thus serve as a marker of increased disease severity. Supporting this, Reddy et al. [24], previously proposed that atrial fibrillation, which develops as a consequence of underlying cardiac disease, could suggest the presence of occult heart failure with preserved ejection fraction. Hence, in patients with severe MR and pEF, atrial fibrillation could carry important prognostic implications.

The modified H2FPEF score provides a holistic assessment by also considering factors such as obesity, hypertension and an advanced age, which are each independently associated with adverse clinical outcomes. For instance, obesity is often accompanied by elevated cardiac output [25], which can exacerbate or contribute synergistically to the volume overload observed in MR. In addition, increased epicardial adipose tissue in obese patients is associated with left atrial dysfunction [26]. In the context of severe MR, such dysfunction may limit the left atrium’s ability to adapt to volume overload, potentially compromising compensatory mechanisms targeted at maintaining ejection fraction [27], and thereby contributing to a poorer prognosis.

Despite the presence of severe MR, its potential chronicity can permit adaptive physiological mechanisms that mask symptomatology in patients, potentially leading to under-recognition of disease severity [5]. Transthoracic echocardiography remains the primary modality for assessing MR and guiding decisions regarding the need for intervention [28]. However, the presence of pEF and the absence of symptoms may complicate clinical assessment [5]. In such situations, the modified H2FPEF score may identify patients at higher risk of adverse outcomes. Whether incorporation of the modified H2FPEF score into clinical decision-making could help identify patients who may benefit from earlier mitral valve intervention or more intensive follow-up warrants prospective investigation. Moreover, the individual components of the modified H2FPEF score may highlight potentially modifiable risk factors. For example, elevated pulmonary artery systolic pressures may be a target for treatment [23] in severe MR and addressed through optimization of guideline-directed medical therapies [29], including the use of diuretics and angiotensin-converting enzyme inhibitors.

Of note, ESRD was more prevalent among patients with high H2FPEF scores (15%) compared with those with intermediate (7.3%) or low (0%) scores, and was independently associated with a higher modified H2FPEF scores. Prior studies have shown that ESRD may contribute to the development or progression of mitral regurgitation through mechanisms such as mitral annular calcification [30]. In addition, ESRD has been associated with macrovascular and myocardial changes, including impaired left ventricular relaxation, that promote the development of HFpEF [31]. These observations suggest that the presence of ESRD may be an important prognostic factor in patients with severe MR and pEF. Hence, incorporating ESRD status into an expanded H2FPEF score, or future models could enhance risk stratification.

There are several notable limitations for our study. First, given the retrospective nature of our study, we could only demonstrate associations rather than causation. Second, our study only makes use of single-center data with a predominantly Asian population, which may limit the generalizability of our findings to the non-Asian or Western populations, as well as to other healthcare systems. Third, we lacked information regarding the exact symptoms experienced by the patients at presentation, which could have granted greater insight into the nature of presentation of patients across modified H2FPEF scores. Fourth, we only had modified H2FPEF scores calculated at the index echocardiographic diagnosis. Hence, we were unable to trend changes in modified H2FPEF scores over time. Fifth, we lacked data on the formal diagnosis of heart failure, which could have confounded the primary composite endpoint. However, this limitation is likely mitigated as we had adjusted for NYHA class, which can act as a surrogate for heart failure status [10]. Sixth, the smaller number of patients in the low modified H2FPEF group and small number of HFH events led to wide confidence intervals reflecting limited statistical precision due to sparse event data. This limitation also precluded Fine–Gray competing-risk analyses involving the low-score group for HFH. We are therefore unable to determine whether a graded with HFH risk exists across the spectrum of H2FPEF scores. Seventh, we lacked data regarding the specific timepoints when patients received mitral valve intervention. This could possibly confound the interpretation of our results, with patients who receive earlier interventions having improved outcomes and vice versa [32,33]. Finally, first HFH was recorded from a single-center database. Accordingly, admissions at other centers may not have been recorded.

The findings of our study should be interpreted as hypothesis-generating rather than confirmatory. They warrant validation in larger, prospective, multi-center cohorts. Furthermore, a future targeted formal discrimination analysis within such studies will help establish whether the modified H2FPEF score provides incremental prognostic value beyond established clinical and echocardiographic markers of severe MR, including PASP and E/e’, thereby clarifying its utility as a risk-stratification tool in this population.

## 5. Conclusions

In conclusion, patients with severe MR and pEF who had high modified H2FPEF scores exhibited a greater prevalence of comorbidities and experienced a greater symptom burden compared to those with lower scores. Malay ethnicity, HLD, DM, ESRD and an increasing LADi were identified as independent variables associated with higher modified H2FPEF scores. Importantly, high or intermediate modified H2FPEF scores were associated with a greater risk of all-cause mortality and first HFH as a composite outcome. These findings suggest that the modified H2FPEF score could serve as a valuable tool for prognostication and risk stratification in patients with severe MR and pEF.

## Figures and Tables

**Table 1:** Baseline and clinical characteristics.

Characteristic	Overall N = 388	Low H2FPEF N = 57 (14.7%)	Intermediate H2FPEF N = 218 (56.2%)	High H2FPEF N = 113 (29.1%)	*p*
Age, mean (SD)	64 (15)	47 (12)	64 (13)	73 (10)	<0.001
Male, n (%)	247 (64%)	41 (72%)	143 (66%)	63 (56%)	0.078
Ethnicity, n (%)					0.12
Chinese	274 (75%)	41 (77%)	157 (78%)	76 (69%)	
Malay	19 (5.2%)	4 (7.5%)	10 (5.0%)	5 (4.5%)	
Indian	49 (13%)	3 (5.7%)	23 (11%)	23 (21%)	
Others	23 (6.3%)	5 (9.4%)	12 (5.9%)	6 (5.5%)	
Height, cm, mean (SD)	163 (10)	168 (8)	163 (10)	161 (9)	<0.001
Weight, kg, mean (SD)	62 (15)	60 (10)	62 (15)	64 (16)	0.2
BMI, kg/m^2^, mean (SD)	23.3 (4.6)	21.2 (2.9)	23.1 (4.6)	24.7 (4.9)	<0.001
Systolic BP, mmHg, mean (SD)	130 (20)	126 (17)	131 (20)	129 (19)	0.2
Diastolic BP, mmHg, mean (SD)	72 (11)	70 (10)	73 (12)	73 (11)	0.2
NYHA Class, n (%)					0.020
1	290 (75%)	45 (79%)	171 (78%)	74 (65%)	
2	66 (17%)	12 (21%)	31 (14%)	23 (20%)	
3–4	32 (8.2%)	0 (0%)	16 (7.3%)	16 (14.7%)	
Medical history, n (%)					
DM	66 (17%)	2 (3.5%)	39 (18%)	25 (22%)	0.008
HTN	209 (54%)	7 (12%)	121 (56%)	81 (72%)	<0.001
HLD	156 (40%)	11 (19%)	94 (43%)	51 (45%)	0.002
Smoking	67 (17%)	15 (26%)	37 (17%)	15 (13%)	0.10
MI	19 (4.9%)	1 (1.8%)	12 (5.5%)	6 (5.3%)	0.6
PCI	16 (4.1%)	2 (3.5%)	11 (5.0%)	3 (2.7%)	0.7
Heart failure	74 (19%)	3 (5.3%)	31 (14%)	40 (35%)	<0.001
PVD	6 (1.5%)	0 (0%)	2 (0.9%)	4 (3.5%)	0.2
Stroke/TIA	37 (9.5%)	3 (5.3%)	21 (9.6%)	13 (12%)	0.4
AF	105 (27%)	0 (0%)	15 (6.9%)	90 (80%)	<0.001
ESRD	33 (8.5%)	0 (0%)	16 (7.3%)	17 (15%)	0.001
Medication history, n (%)					
Beta-Blocker	126 (32%)	7 (12%)	59 (27%)	60 (53%)	<0.001
ACEI/ARB	82 (21%)	5 (8.8%)	46 (21%)	31 (27%)	0.019
DM Medications	29 (7.5%)	1 (1.8%)	16 (7.3%)	12 (11%)	0.049
Diuretics	74 (19%)	1 (1.8%)	37 (17%)	36 (32%)	<0.001
CCB	56 (14%)	1 (1.8%)	39 (18%)	16 (14%)	0.008
Statins	119 (31%)	5 (8.8%)	67 (31%)	47 (42%)	<0.001
Anti-platelets	73 (19%)	6 (11%)	43 (20%)	24 (21%)	0.10
Anti-coagulants	50 (13%)	2 (3.5%)	12 (5.5%)	36 (32%)	<0.001
Mitral valve intervention, n (%)	119 (31%)	21 (37%)	73 (33%)	25 (22%)	0.057

BMI body mass index; BP blood pressure; DM diabetes mellitus; HTN hypertension; HLD hyperlipidemia; MI myocardial infarction; PCI percutaneous coronary intervention; PVD peripheral vascular disease; TIA transient ischemic attack; AF atrial fibrillation; ESRD end-stage renal disease; ACEI angiotensin-converting enzyme inhibitor; ARB angiotensin II receptor blocker; CCB calcium channel blockers.

**Table 2:** Echocardiographic variables.

Variable	Low H2FPEF N = 57 (14.7%)	Intermediate H2FPEF N = 218 (56.2%)	High H2FPEF N = 113 (29.1%)	*p*
LVEDD, mm	55 (7)	54 (7)	54 (8)	0.7
LVEDDi, mm/m^2^	32.9 (3.4)	33.0 (5.0)	32.2 (5.1)	0.4
LVESD, mm	33 (6)	34 (6)	35 (7)	0.4
LVESDi, mm/m^2^	20.2 (3.3)	20.4 (4.0)	20.8 (4.2)	0.6
LVEDV, ml	148 (40)	146 (45)	143 (47)	0.7
LVEDVi, ml/m^2^	88 (21)	88 (25)	85 (26)	0.5
LVESV, ml	47 (20)	48 (22)	52 (25)	0.4
LVESVi, ml/m^2^	28 (12)	29 (12)	31 (14)	0.4
LVEF, %	62.9 (5.4)	62.7 (4.9)	61.3 (5.9)	0.028
PASP, mmHg	26 (14)	42 (20)	54 (18)	<0.001
IVSd, mm	9.18 (1.99)	10.04 (2.09)	10.42 (1.78)	<0.001
IVSs, mm	13.75 (2.80)	14.56 (2.68)	14.78 (2.63)	0.072
LVPWD, mm	9.04 (1.67)	9.69 (1.77)	10.18 (1.81)	<0.001
LVPWS, mm	15.07 (2.92)	15.02 (2.63)	15.28 (2.56)	0.5
RWT	0.34 (0.07)	0.36 (0.09)	0.39 (0.10)	<0.001
LVmass, g	191 (59)	209 (66)	218 (67)	0.061
Indexed LVmass, g/m^2^	114 (30)	125 (33)	129 (35)	0.030
LADi, mm/m^2^	24 (5)	28 (7)	32 (9)	<0.001
RV peak pressure gradient, mmHg	17 (10)	31 (16)	40 (16)	<0.001
MR EROA, cm^2^	0.57 (0.38)	0.60 (0.60)	0.49 (0.35)	0.3
RV, ml	98 (47)	105 (67)	92 (52)	0.2
RF, %	61 (11)	62 (14)	62 (12)	0.9

EROA effective regurgitant orifice area; LVEDDi indexed left ventricular end-diastolic diameter; LVEDVi indexed left ventricular end-diastolic volume; LVESDi indexed left ventricular end-systolic diameter; LVESVi indexed left ventricular end-systolic volume; IVSd interventricular septal thickness at diastole; IVSs interventricular septal thickness at systole; LVEDD left ventricular end-diastolic diameter; LVEDV left ventricular end-diastolic volume; LVEF left ventricular ejection fraction; LVESD left ventricular end-systolic diameter; LVESV left ventricular end-systolic volume; LVmass left ventricular mass; LVPWD left ventricular posterior wall thickness at diastole; LVPWS left ventricular posterior wall thickness at systole; MR mitral regurgitation; PASP pulmonary artery systolic pressure; RF regurgitant fraction; RV regurgitant volume; RV right ventricle; RWT relative wall thickness.

**Table 3:** Ordinal logistic analysis to assess association of variables with the modified H2FPEF score.

Variable	Univariate Model		Multivariate Model	
	Odds Ratio, 95% CI	*p*	Odds Ratio, 95% CI	*p*
Male Sex	0.64 (0.45 to 0.93)	0.018	0.86 (0.58 to 1.30)	0.5
Ethnicity		0.003		0.004
Chinese	1.00 (ref)		1.00 (ref)	
Malay	2.39 (1.39 to 4.12)		2.47 (1.42 to 4.29)	
Indian	0.71 (0.30 to 1.65)		0.70 (0.29 to 1.68)	
Others	0.59 (0.28 to 1.25)		0.66 (0.30 to 1.44)	
DM	2.04 (1.31 to 3.21)	0.002	1.73 (1.06 to 2.82)	0.027
HLD	1.67 (1.17 to 2.38)	0.005	1.97 (1.35 to 2.89)	<0.001
ESRD	2.99 (1.63 to 5.54)	<0.001	2.15 (1.07 to 4.33)	0.032
Smoking	0.67 (0.42 to 1.06)	0.085		
History of stroke/TIA	1.71 (0.96 to 3.08)	0.071		
NYHA class		0.007		0.10
1	1.00 (ref)		1.00 (ref)	
2	1.30 (0.80 to 2.12)		1.15 (0.70 to 1.90)	
3–4	2.78 (1.44 to 5.39)		2.16 (1.07 to 4.37)	
LVEF	0.95 (0.92 to 0.99)	0.007	0.99 (0.96 to 1.03)	0.7
LVEDVi	0.99 (0.99 to 1.00)	0.092		
LADi	1.09 (1.06 to 1.11)	<0.001	1.08 (1.05 to 1.11)	<0.001
LV mass index	1.01 (1.00 to 1.01)	0.003	1.00 (1.00 to 1.01)	0.3
MR EROA	0.76 (0.55 to 1.06)	0.10		

CI confidence interval; ESRD end-stage renal disease; DM diabetes mellitus; HLD hyperlipidemia; LADi indexed left atrial diameter; LV left ventricular; LVEDVi indexed left ventricular end-diastolic volume; LVEF left ventricular ejection fraction; MR EROA mitral regurgitation effective regurgitant orifice area; NYHA New York heart association; TIA transient ischemic attack.

## Data Availability

The raw data supporting the conclusions of this article will be made available by the authors on request.

## Supplementary Materials

The following supporting information can be downloaded at https://www.mdpi.com/article/10.3390/jcdd13090439/s1, Supplemental Figure S1: Flowchart for data inclusion; Supplemental Table S1: Univariate and Multivariate Cox regression for the primary composite endpoint, Supplemental Table S2: Multivariate Cox regression for the primary composite outcome and secondary outcome of all-cause mortality (Sensitivity analysis excluding patients who underwent mitral valve intervention), Supplemental Table S3: Univariate and Multivariate Cox regression for the secondary outcome of all-cause mortality, Supplemental Table S4: Fine–Gray Model for comparative risk of heart failure admissions in patients with high vs. intermediate modified H2FPEF scores.

## Abbreviations

The following abbreviations are used in this manuscript:

MR	Mitral Regurgitation
(LV)EF	(Left Ventricular) Ejection Fraction
PASP	Pulmonary Artery Systolic Pressure
HFH	Heart Failure Hospitalization
LADi	Indexed Left Atrial Diameter
