# Supplementary material for: Prognostic Significance of the Modified H2FPEF Score in Patients with Severe Primary Mitral Regurgitation
Source: J Cardiovasc Dev Dis. 2026 Sep 7;13(9):439. doi: 10.3390/jcdd13090439 (PMC13607464; doi:10.3390/jcdd13090439)
Supplement: Supplementary file 1 [file jcdd-13-00439-s001.zip › jcdd-4429040-supplementary.pdf]

Supplementary Information

Supplemental Figure S1: Flowchart for data inclusion

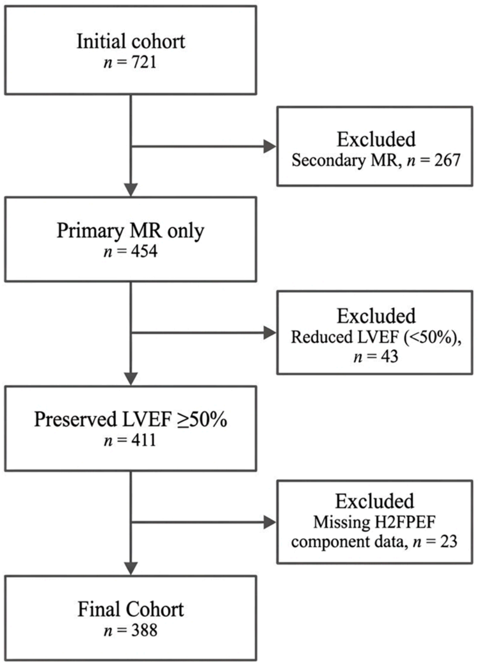

Supplemental Table S1: Univariate and Multivariate Cox regression for the Primary Composite Endpoint

**Variable**	**No. of events**	**Univariate Model***		**Multivariate Model^***	
		HR, 95% CI	*P*	HR, 95% CI	*P*
**Modified H_2FPEF score**			<0.001		<0.001
Low (0-1)	2	1.00 (ref)		1.00 (ref)	
Intermediate (2-5)	49	7.24 (1.76-29.8)		6.71 (1.62-27.9)	
High (6-9)	40	12.60 (3.03-52.1)		9.17 (2.15-39.1)	

CI confidence interval; *HR* hazard ratio

^Adjusted for sex, diabetes mellitus, hyperlipidemia, NYHA classification, end-stage renal disease, left ventricular ejection fraction, indexed left ventricular end-systolic diameter

*Proportional hazards assumption: univariate model (*P* = 0.48); multivariate model (*P* = 0.17)

Supplemental Table S2: Multivariate Cox regression for the primary composite outcome and secondary outcome of all-cause mortality (Sensitivity analysis excluding patients who underwent mitral valve intervention)

**Variable**		**Primary composite outcome^***			**All-cause mortality^***	
	No. of events	HR, 95% CI	*P*	No. of events	HR, 95% CI	*P*
**Modified H_2FPEF score**			0.002			0.007
Low (0-1)	1	1.00 (ref)		1	1.00 (ref)	
Intermediate (2-5)	37	9.42 (1.28-69.30)		35	8.87 (1.20-65.30)	
High (6-9)	35	12.10 (1.61-90.80)		28	9.13 (1.21-69.00)	

CI confidence interval; *HR* hazard ratio

^Adjusted for sex, diabetes mellitus, hyperlipidemia, NYHA classification, end-stage renal disease, left ventricular ejection fraction, indexed left ventricular end-systolic diameter<br>*Proportional hazards assumption: composite outcome (*P* = 0.14); all-cause mortality (*P* = 0.064)

Supplemental Table S3: Univariate and Multivariate Cox regression for the secondary outcome of all-cause mortality

**Variable**	**No. of events**	**Univariate Model***		**Multivariate Model^***	
		HR, 95% CI	*P*	HR, 95% CI	*P*
**Modified H_2FPEF score**			0.009		0.004
Low (0-1)	2	1.00 (ref)		1.00 (ref)	
Intermediate (2-5)	41	5.83 (1.41-24.1)		5.81 (1.39-24.3)	
High (6-9)	30	8.60 (2.06-36.0)		6.70 (1.55-29.0)	

CI confidence interval; *HR* hazard ratio

^Adjusted for sex, diabetes mellitus, hyperlipidemia, NYHA classification, end-stage renal disease, left ventricular ejection fraction, indexed left ventricular end-systolic diameter

*Proportional hazards assumption: univariate model (*P* = 0.31); multivariate model (*P* = 0.12)

Supplemental Table S4: Fine-Gray Model for Comparative Risk of Heart Failure Admissions in Patients with High vs Intermediate Modified H_2FPEF scores

**Variable**	**Adjusted HR^ (95% CI)**	***P***
High modified H2FPEF score	1.79 (0.89-3.58)	0.10
Male sex	0.94 (0.43-2.03)	0.87
DM	1.02 (0.39-2.67)	0.97
HLD	1.36 (0.60-3.12)	0.46
ESRD	1.23 (0.35-4.31)	0.75
LVEF	0.97 (0.88-1.06)	0.45
NYHA group		
1	Reference	
2	1.63 (0.61-4.39)	0.33
3–4	6.79 (2.87-16.02)	<0.001
Indexed left ventricular end-systolic diameter	1.04 (0.97-1.11)	0.31

ESRD end-stage renal disease; *CI* confidence interval; *DM* diabetes mellitus; *HLD* hyperlipidemia; *HR* hazard ratio; *LVEF* left ventricular ejection fraction; *NYHA* New York heart association

^Adjusted for sex, diabetes mellitus, hyperlipidemia, NYHA classification, end-stage renal disease, indexed left ventricular end-systolic diameter
